# A combined transcriptomics and proteomics approach reveals S100A4 as a potential biomarker for Graves’ orbitopathy

**DOI:** 10.3389/fgene.2024.1342205

**Published:** 2024-09-18

**Authors:** Chiaw-Ling Chng, Oi Fah Lai, Lay-Leng Seah, Kai-Ling Yong, Yvonne Hsi-Wei Chung, Rochelle Goh, Che Kang Lim

**Affiliations:** ^1^ Department of Endocrinology, Singapore General Hospital, Singapore, Singapore; ^2^ Department of Clinical and Translational Research, Singapore General Hospital, Singapore, Singapore; ^3^ Oculoplastic Department, Singapore National Eye Centre, Singapore, Singapore

**Keywords:** Graves’ ophthalmology, Graves’ orbitopathy, transcriptome, tear biomarkers, S100A4

## Abstract

**Background:**

There are no reliable biomarkers to identify Graves’ disease patients who will develop severe Graves’ orbitopathy (GO). We hypothesize that integrating various omics platforms can enhance our understanding of disease mechanisms and uncover potential biomarkers. This study aimed to (1) elucidate the differential gene expression profile of orbital fibroblasts in GO during early adipogenesis to better understand disease mechanisms and (2) compare tear protein profiles from our earlier study and the transcriptome profiles of orbital fibroblasts (OFs) to identify possible biomarkers of the disease.

**Methods:**

OFs were grown from orbital adipose tissue obtained from nine GO patients (three for discovery and six for validation experiments). Total RNA was extracted from OFs on day 0 as the baseline for each sample and from differentiated OFs on days 4 and 8. Protein–protein interaction (PPI) analysis and functional enrichment analysis were also carried out. The differentially expressed genes (DEGs) from the RNA sequencing experiments were then compared to the full tear proteome profile from the author’s previous study, which examined the tear protein changes of GO patients based on fold change > 1.6 or < −1.6. FDR < 0.05 was applied within all datasets. Further validation of S100 calcium-binding protein A4 (S100A4) downregulation in GO was performed via quantitative real-time PCR (qPCR).

**Results:**

The whole transcriptomic analysis revealed 9 upregulated genes and 15 downregulated genes in common between the discovery and validation experiments. From the PPI network analysis, an interaction network containing six identified DEGs (ALDH2, MAP2K6, MT2A, SOCS3, S100A4, and THBD) was observed. The functional enrichment network analysis identified a set of genes related to oxysterol production. S100A4 was found to be consistently downregulated in both our transcriptome studies and the full-tear proteome profile from the author’s previous study.

**Conclusion:**

Our study identified several DEGs and potential gene pathways in GO patients, which concurred with the results of other studies. Tear S100A4 may serve as a biomarker for the propensity to develop thyroid eye disease (TED) in patients with autoimmune thyroid disease (AITD) before clinical manifestation and should be confirmed in future studies.

## Introduction

Graves’ orbitopathy (GO) is the most common extrathyroidal manifestation of Graves’ disease (GD), affecting up to half the patients, with 5% progressing to sight-threatening disease (Bahn, 2010). Despite advances in treatment, a significant proportion of patients are undiagnosed, resulting in long-term debilitating consequences, such as diplopia and exposure keratopathy, severely impairing vision and quality of life. Hence, early diagnosis and treatment form the cornerstone of the management of GO. Currently, there are no reliable biomarkers to identify GD patients who will develop severe GO. Orbital fibroblasts (OFs) play a pivotal role in the pathogenesis of GO, functioning both as a major target of inflammatory cytokines released by infiltrating immune cells and as active participants in the perpetuation of orbital disease (Dik et al., 2016). Orbital tissue expansion and fibrosis, central consequences of the pathogenic processes, result from the proliferation, extracellular matrix production, and differentiation of OFs into adipocytes and myofibroblasts. The molecular mechanisms underlying orbital adipogenesis in GO are not well understood, and several studies have explored transcriptome profiling via RNA sequencing during orbital fibroblast adipogenesis in GO patients to elucidate potential treatment targets (Lee et al., 2018; Kim et al., 2021; Bai et al., 2022). Recent studies have also shown the involvement of lacrimal glands in the pathogenesis of ocular surface damage in GO (Huang et al., 2014; Eckstein et al., 2004). Tear sampling provides a convenient and non-invasive method of analyzing an accessible body fluid for the investigation of potential biomarkers in ocular diseases. Previously, the author demonstrated different tear profiles in patients with different severity levels of GO, which suggests the role of the tear proteome as a potential biomarker of GO development and progression in patients with autoimmune thyroid disease (AITD) (Chng et al., 2018). This study aimed to (1) investigate the transcriptome profiles of orbital fibroblasts during early adipogenesis and (2) compare tear protein profiles from our earlier study and the transcriptome profiles of orbital fibroblasts to identify possible common molecular markers of the disease.

## Methodology

### Patient recruitment

The study was approved by the SingHealth Centralized Institutional Review Board. A total of nine patients with GO who underwent orbital decompression or eyelid mullerectomy (one case) were recruited for this study. All patients with GO were managed by an ophthalmologist. The diagnosis of GO was made based on diagnostic criteria defined by Bartley and Gorman (1995), i.e., GO is present if eyelid retraction occurs in association with thyroid dysfunction, exophthalmos, optic nerve dysfunction, or extraocular muscle involvement, and other confounding causes such as idiopathic orbital inflammation are excluded. All the patients with GO were euthyroid and had inactive disease at the time of surgery. Subjects who were smokers, diabetic, and had a recent (<3 months) intake of steroids, immunomodulatory agents, or orbital radiation were also excluded. Smoking is known to increase the incidence and severity of thyroid eye disease (TED) and induce numerous gene expression changes (Bahn, 2010). Smoking also affects the adipogenesis of orbital fibroblasts (Chng et al., 2014). Steroids, diabetes, immunomodulatory agents, and orbital radiation are potential confounding factors that may influence adipogenesis, inflammation, and overall transcriptional activity in orbital adipose tissues. For the discovery phase, orbital adipose tissue from three GO cases (age 48–68 years, all female patients) recruited between 2016 and 2017 was used for the experiments (Table 1). For the validation phase, orbital adipose tissue from six GO cases (age 27–61 years, five female and one male patient) recruited between 2020 and 2022 was used (Table 1).

**TABLE 1:** Clinical characteristics of cases recruited for the study.

	Discovery set			Validation set					
Clinical information	Case 1	Case 2	Case 3	Case 1	Case 2	Case 3	Case 4	Case 5	Case 6
Age (years)	48	68	66	27	61	40	33	30	60
Gender	F	F	F	F	F	F	M	F	F
GD duration (years)	10	0.5	9.5	4	25	4	0^a^	2	3
TED duration (years)	10	0.5	9.5	3	1	3	7	1.5	2
Main treatment for thyrotoxicosis	RAI	CMZ	CMZ	CMZ	T	RAI	Nil	CMZ	RAI
Prior treatment for TED	Nil	S	Nil	S	S	S	Nil	Nil	S
Medical hx	HTN	Nil	HTN/HLD	Nil	Nil	Nil	Nil	Nil	HTN/HLD
Dysthyroid optic neuropathy	No	No	No	No	No	No	No	No	No
CAS (0–10)	0	0	2	0	2	1	0	0	0
Surgery	BOD	ROD	BOD	BOD	ROD	EM	BOD	LOD	LOD
TRAB (0–1.5 IU/L)	0.5	4.3	12.8	>40	4.5	26.4	2.02	37.1	16

^a^ Case 4 (validation set) is a euthyroid GO case.

RAI, radioiodine; CMZ, carbimazole; T, thyroidectomy; S, steroid; HTN, hypertension; HLD, hyperlipidemia; BOD, bilateral orbital decompression; ROD, right-orbital decompression; EM, eyelid mullerectomy; LOD, left-orbital decompression; TRAb, TSH receptor antibody.

### Orbital fibroblast cultures

**FIGURE 1:**
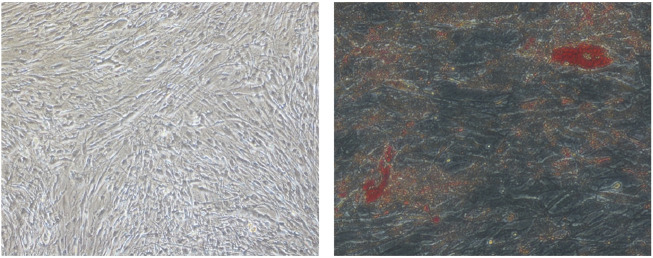
Digital photographs (at × 20 magnification) of OFs from GO subjects showing adipogenesis. (Left) D18 undifferentiated OFs. (Right) D18 differentiated OFs. The red spots are intracellular lipid droplets stained with Oil Red O.

OFs were grown from the orbital adipose tissue obtained. The excised fat was minced into 0.1 cm × 0.1 cm pieces and placed in plastic culture flasks in M199 (Gibco, Life Technologies) supplemented with 10% fetal bovine serum (FBS), gentamycin (20 ug/mL), and penicillin/streptomycin (100 u/mL), allowing OFs to emerge from the tissue as described previously (Valyasevi et al., 1999). Fibroblasts were expanded over a period of 3–4 weeks till sufficient cells were obtained. When sufficient cells were available for inducing lipid accumulation, orbital fibroblasts were seeded at a density of 2.1 × 10^6^ cells per mL into T75 culture flasks. When the cells approached confluence, an adipogenic induction medium (AIM) comprising IMDM supplemented with 10% FBS, 0.528 mM IBMX, 0.033 uM biotin, 0.001 mM dexamethasone, 0.2 mM indomethacin, and 0.174 uM insulin was used to induce lipid accumulation in orbital fibroblasts. The medium was changed every 2 days to an adipogenic maintenance medium (IMDM with 10% FBS supplemented with 0.174 uM insulin). The cycling of induction and maintenance media was carried out until D18. Cells differentiated until D18 in 6-well culture flasks were stained with Oil Red O on D18 to visualize lipid accumulation and adipocyte morphology (Figure 1). For this study, cultures from D0 to D4 and D0 to D8 were used for both the discovery and validation phases. These time points were chosen to focus on the gene expression related to early adipogenesis.

### Whole-transcriptome sequencing (total RNA sequence)

Total RNA was extracted from OFs on day 0 as the baseline for each sample and from differentiated OFs on days 4 and 8 using an RNeasy Kit (QIAGEN). Total RNA libraries were prepared using the Illumina TruSeq Kit and sequenced on the Illumina NovaSeq 6000 Sequencing Platform (Illumina, San Diego, CA, United States). Paired-end reads (150 bp) were mapped to the GRCh38 reference human genome. Output BCL files were converted to FASTQ files and demultiplexed using bcl2fastq v.2.20 from Illumina. Overall, approximately 40 million reads per sample were obtained.

### Comparative tear protein samples

The differentially expressed genes (DEGs) from the RNA sequencing experiments were then compared to the full tear proteome profile from the author’s previous studies, which examined the tear protein changes of GO patients with increasing severity compared to normal controls (Chng et al., 2018).

### Quantitative real-time PCR for S100A4

Total RNA was extracted from OFs on day 0 as the baseline for each sample and from differentiated OFs from patients with GO on days 4 and 8 using the RNeasy Mini RNA Isolation and QIAshredder Kits (QIAGEN). A 15-min on-column DNase digestion was carried out to eliminate genomic DNA as outlined in the kit manual. Then, 1 ug of RNA was reverse-transcribed using a High-Capacity cDNA Reverse Transcription Kit (Applied Biosystems, Foster City, United States). Quantitative real-time PCR (qPCR) was performed in duplicate using reagents from the GoTaq qPCR Kit (Promega) in 96-well hard-shell PCR plates (Bio-Rad). The primers used for S100 calcium-binding protein A4 (S100A4) analysis were as follows: forward primer, TCT​TGG​TTT​GAT​CCT​GAC​TGC​T; reverse primer, ACT​TGT​CAC​CCT​CTT​TGC​CC. Cycling conditions were induced using the Bio-Rad Real-Time Thermal Cycler. qPCR cycling was carried out according to the manufacturer’s guidelines: 2 min of initial activation at 95°C, 40 cycles of denaturation at 95°C for 15 s, and annealing and extension for 1 min at 60°C. The relative gene expression of S100A4 was analyzed using the comparative CT (2^−^ôô ^CT^) normalized against housekeeping gene β-actin.

### Bioinformatics and statistical analysis

**FIGURE 2:**
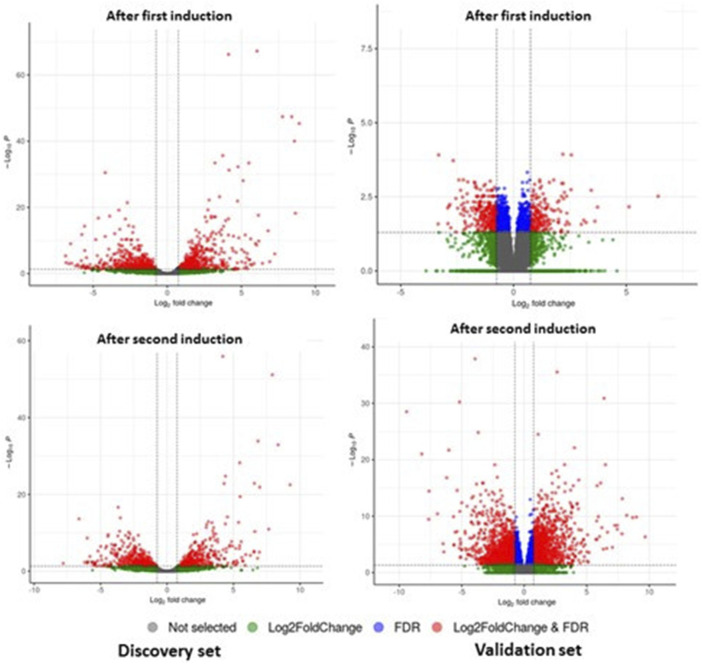
DEGs of mRNA between patients with GO and controls from discovery and validation phase experiments for first induction (D0–D4) and second induction (D0–D8). The volcano plots display the fold changes and *p*-values of differential mRNA expression in patients with GO compared to normal controls. Based on the relationship between fold change and statistical significance, subsets of mRNAs were isolated. The cut-off for logFC is 1.6. The red points represent the upregulated mRNAs with statistical significance *p* < 0.05, while the green dots represent the significantly downregulated expression.

Sequencing read quality was assessed using FASTQC software, and low-quality bases were removed from individual reads using the cutadapt tool. Reads were mapped to genes through alignment to the human reference transcriptome (GENCODE version 38), and gene expression levels were quantified using Salmon Software. Differential gene expression analysis was conducted using DESeq2 (Love et al., 2014) by comparing baseline (D0) versus “after first induction” (D0–D4) and baseline (D0) versus “after second induction” (D0–D8). The statistical significance of differences was assessed using the Student’s *t*-test. Differentially expressed mRNAs were identified by fold change (FC) > 1.6 or FC < −1.6 and false discovery rate (FDR) < 0.05 (Figure 2).

In addition to the gene-level analysis, protein–protein interaction (PPI) analysis and functional enrichment analysis were carried out via Search Tool for the Retrieval of Interacting Genes/Proteins (STRING) database v12.0 (Szklarczyk et al., 2023) for the final enriched DEGs. Functional enrichment analysis was repeated using Database for Annotation, Visualization and Integrated Discovery (DAVID) (Sherman et al., 2022) to reconfirm the result.

## Results

Whole-transcriptomic analysis identified 672 DEGs (FC ≥ 1.6 or FC < −1.6; FDR <0.05) in the discovery set from both induction cycles (D0–D4 and D0–D8) (Supplementary Table S1). On the other hand, 112 significantly differentially expressed genes were observed in the validation set (Supplementary Table S2) that were related to orbital adipogenesis. We next examined the shared (intersection) DEGs between 2 sets and found 24 DEGs during the early adipogenesis of OFs from GO patients after filtering with maintenance controls. The differential expression remained consistent in both the discovery and validation phase experiments. Of these, 9 genes were upregulated, and 15 were downregulated (Table 2).

**TABLE 2:** DEGs in orbital fibroblasts derived from patients with GO when compared to normal controls. OFs were induced from D0 to D4 and from D0 to D8 for both discovery and validation phase experiments. FDR < 0.05; log2FC > 0.678 or < −0.678 (FC > 1.6 or < −1.6).

		Discovery set				Validation set			
		D0 to D4		D0 to D8		D0 to D4		D0 to D8	
Gene	Gene description	Log_2_FC	FDR	Log_2_FC	FDR	Log_2_FC	FDR	Log_2_FC	FDR
PDK4	Pyruvate dehydrogenase kinase 4	7.785	3.965E-48	7.920	7.459E-52	1.748	9.271E-03	6.110	1.363E-16
MAP2K6	Mitogen-activated protein kinase 6	5.036	5.862E-08	4.221	2.448E-06	1.396	5.048E-03	4.361	4.049E-17
HSD11B1	Hydroxysteroid 11-beta dehydrogenase 1	3.650	2.597E-08	3.811	2.556E-08	2.130	4.135E-02	4.865	1.068E-05
SLC19A2	Solute carrier family 19 member 2	3.043	9.190E-08	3.366	2.493E-07	1.396	3.663E-03	3.292	1.480E-16
MT2A	Metallothionein 2A	3.036	3.369E-08	3.237	2.302E-09	1.986	8.290E-03	3.467	2.111E-08
SLC16A9	Solute carrier family 16 member 9	2.612	2.539E-04	1.965	1.154E-02	1.202	2.806E-02	1.786	6.440E-06
GPAM	Glycerol-3-phosphate acyltransferase, mitochondrial	2.336	1.135E-08	2.125	4.487E-05	0.684	6.285E-03	2.785	3.231E-11
SAT1	Spermidine/spermine N1-acetyltransferase 1	2.034	2.340E-09	2.129	3.027E-08	1.135	3.242E-03	3.244	4.487E-15
ALDH2	Aldehyde dehydrogenase 2 family (mitochondrial)	1.415	3.725E-04	1.611	1.412E-04	0.767	7.473E-03	2.375	3.982E-12
SFRP4	Secreted frizzled related protein 4	−1.230	7.598E-04	−2.327	4.437E-04	−2.212	1.340E-02	−1.950	3.100E-02
AMACR	Alpha-methylacyl-CoA racemase	−1.668	8.859E-04	−1.465	1.804E-02	−0.729	4.990E-02	−0.678	4.501E-02
LRRN4CL	LRRN4 C-terminal-like	−1.895	2.703E-04	−2.006	1.456E-02	−0.863	6.527E-03	−1.318	3.826E-04
GALNT12	Polypeptide N-acetylgalactosaminyltransferase 12	−2.030	3.600E-03	−1.836	2.888E-02	−0.919	1.867E-02	−0.867	1.485E-03
IER2	Immediate early response 2	−2.154	4.163E-04	−1.942	2.854E-03	−0.834	4.135E-02	−1.361	5.404E-05
ARHGAP28	Rho GTPase activating protein 28	−2.158	6.247E-03	−2.367	9.543E-03	−1.257	1.378E-02	−1.397	1.675E-03
SOCS3	Suppressor of cytokine signaling 3	−2.188	1.231E-02	−2.776	1.114E-03	−0.886	1.308E-02	−1.728	7.384E-07
ECM2	Extracellular matrix protein 2	−2.400	1.472E-03	−1.768	4.824E-02	−1.731	1.032E-03	−2.335	2.957E-04
PSG1	Pregnancy-specific beta-1-glycoprotein 1	−2.711	2.877E-07	−3.619	2.070E-06	−2.214	4.089E-02	−5.081	4.356E-06
KCND3	Potassium voltage-gated channel subfamily D member 3	−2.721	5.700E-04	−2.492	1.345E-02	−1.598	4.283E-02	−2.116	2.117E-03
HSD3B7	Hydroxy-delta-5-steroid dehydrogenase, 3 beta-, and steroid delta-isomerase 7	−2.762	8.295E-04	−2.888	7.955E-04	−0.763	2.153E-02	−1.913	2.587E-06
EGR2	Early growth response 2	−2.809	3.338E-02	−2.814	4.636E-02	−3.344	2.599E-02	−3.930	3.478E-03
S100A4	S100 calcium-binding protein A4	−3.272	1.488E-09	−2.421	9.775E-06	−0.786	1.649E-02	−2.328	3.035E-07
THBD	Thrombomodulin	−3.616	1.379E-06	−4.135	1.593E-06	−1.103	3.009E-02	−2.492	6.021E-10
IER3	Immediate early response 3	−4.744	9.205E-03	−5.642	4.487E-03	−1.118	7.510E-03	−2.234	4.073E-08

### PPI network associated with the early adipogenesis in GO

**FIGURE 3:**
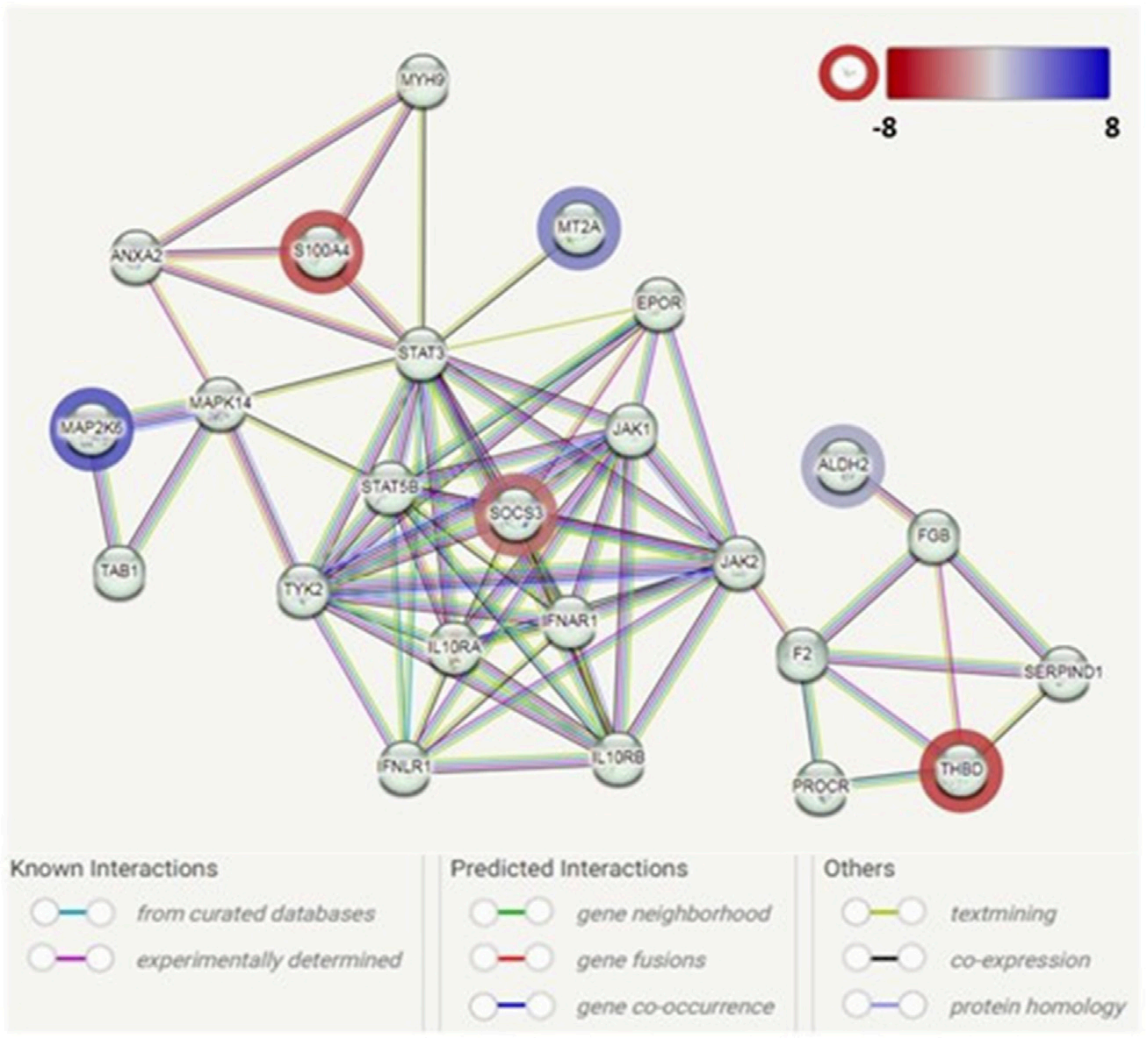
Protein–protein interaction network analysis among the identified genes associated with adipogenesis in GO. Each node represents a protein product. The node highlighted in red indicates downregulated genes/proteins. The node highlighted in blue indicates upregulated genes/proteins. Lines denote protein–protein interactions: light-blue lines represent interactions from curated databases; pink lines represent experimentally determined interactions; green lines represent interactions predicted by gene neighborhood; red lines represent interactions predicted by gene fusions; blue lines represent interactions predicted by gene co-occurrence; yellow lines represent interactions by text mining; black lines represent interactions by co-expression; and purple lines represent interactions by protein homology.

Based on the 24 DEGs listed as input, the main PPI network (Figure 3) associated with the early adipogenesis in GO containing the seed proteins and their neighbors’ interactome was constructed. All the interactions between them were derived from all available active interaction sources in STRING. A medium-to-high level of confidence (score ≥ 0.50) was set. In addition, support from at least two different sources is required.

The PPI network analysis observed an interaction network containing six identified DEGs (*ALDH2*, *MAP2K6*, *MT2A*, *SOCS3*, *S100A4*, and *THBD*). The other nodes of the network mainly contain genes/proteins related to autoinflammatory/autoimmunity, such as JAK1, STAT3, STAT5B, IL10RA, and IL10RB, with *SOCS3* as a hub. Additionally, genes/protein nodes associated with lipid metabolism (e.g., F2 and FGB) were detected and connected with the detected DEG, *ALDH2*.

In addition, through the functional enrichment network analysis in the STRING database, we identified a set of genes—HSD11B1, HSD3B7, and AMACR—that were associated with pathways related to oxysterol production (FDR = 0.0248). The finding was further confirmed using DAVID (Fold enrichment was 31.20, *p*-value: 3.54E-03).

### Comparative transcriptomics and tear proteomic profile analysis

**FIGURE 4:**
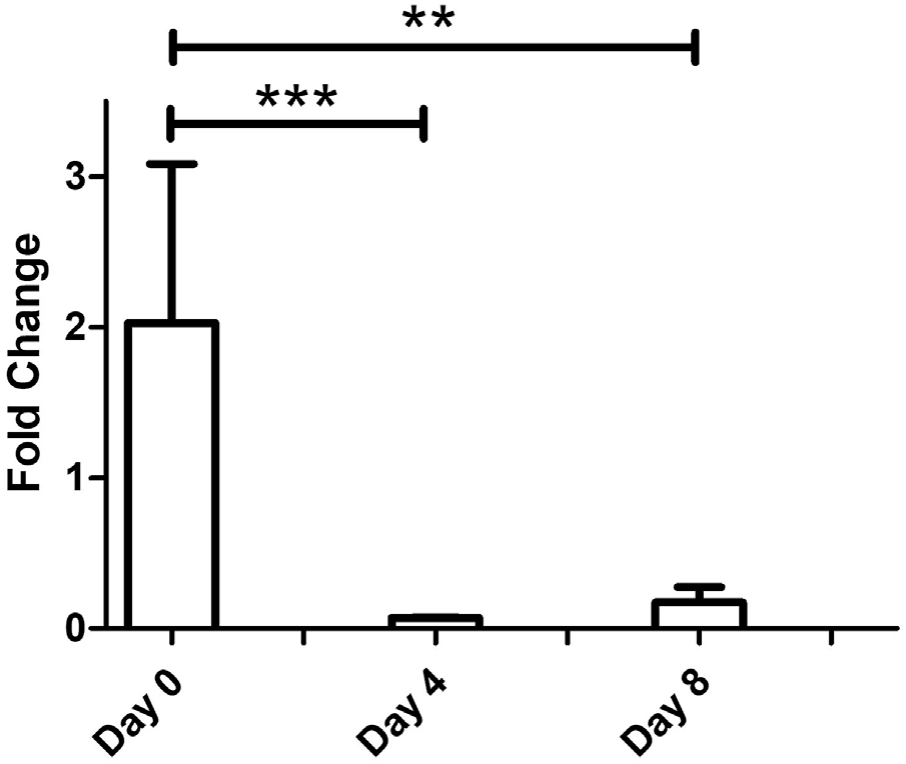
Downregulation of S100A4 relative gene expression in GO patients on days 0, 4, and 8, as measured by qPCR (*p < 0.05, **p < 0.01, and ***p < 0.001).

Next, a comparative transcriptomics and tear proteomic expression profile analysis was performed to identify potential tear marker(s) linked with GO. We compared the transcriptomics expression profile of all 68 tear proteins (24 up and 44 down) that were identified from TED/ GO patients previously (Chng et al., 2018). A standardized filtering cut-off, including consistent expression pattern, fold change >1.6 or < −1.6, and FDR < 0.05, was applied across all datasets (transcriptomics discovery sets, transcriptomics validation sets, and tear proteome sets). Based on the stringent filtering strategy, only one marker, S100A4, was found to be consistently downregulated (Table 3). The downregulation of S100A4 expression during early adipogenesis in orbital fibroblasts in GO was also confirmed by qPCR analysis in the current study, with a significantly reduced fold change in this gene expression on D4 and D8 compared to D0 fibroblast cultures while exposed to the adipogenic medium (Figure 4).

**TABLE 3:** Comparison of DEGs from the RNA sequencing experiments to the full tear proteome profile. FDR < 0.05; Log_2_FC > 0.678 or < −0.678 (FC > 1.6 or < −1.6).

		Discovery set				Validation set				Tear proteome	
		After 1st cycle stimulation		After 2nd cycle stimulation		After 1st cycle stimulation		After 2nd cycle stimulation			
Gene	Gene description	Log_2_FC	FDR	Log_2_FC	FDR	Log_2_FC	FDR	Log_2_FC	FDR	Log_2_FC	FDR
S100A4	S100 calcium-binding protein A4	−3.272	1.488E-09	−2.421	9.775E-06	−2.328	3.035E-07	−0.786	1.649E-02	−1.591	2.90E-02

## Discussion

Our study investigated DEG profiles during the early adipogenesis of OFs derived from patients with GO. These expression profiles were consistent between the discovery and validation experiments conducted using different cohorts of patients recruited over different time periods. Several of the gene expression patterns were noted in other studies related to GO. Pyruvate dehydrogenase kinase (PDK) enzymes trigger the switch from oxidative phosphorylation to cytoplasmic glycolysis, which plays a role in immune processes by promoting cell proliferation and strengthening antioxidant defense (Stacpoole, 2017). PDK2 overexpression has been found to promote perimysial orbital fibroblast proliferation in GO via Akt signaling (Ma et al., 2020). On the other hand, PDK4 has been shown to maintain the stability of HIF-1α (Ma et al., 2023). Notably, hypoxia-dependent HIF-1 activation was found to impact tissue remodeling in GO and may play a role in the worsening of GO due to smoking (Chng et al., 2014; Görtz et al., 2016).

Mitogen-activated protein (MAP2K6/MKK6), 11β-hydroxysteroid dehydrogenase 1 (11β-HSD1), and aldehyde dehydrogenase 2 (ALDH2) play important roles in adipogenesis. The expression of MKK6(Glu) in fibroblasts stimulates extensive p38-dependent adipocyte conversion in the absence of hormonal stimulation and strongly promotes adipogenesis both in 3T3-L1 fibroblasts and NIH-3T3 cells (Engelman et al., 1999). 11β-HSD1 is a bidirectional enzyme that interconverts inactive cortisone and active cortisol, thereby increasing local glucocorticoid bioavailability (Tomlinson et al., 2004). The induction of 11β-HSD1 activity and expression by inflammatory cytokines (TNF and IL-6) may enhance orbital adipogenesis and is pivotal in the regulation of local inflammatory response (Tomlinson et al., 2010). ALDH2 activation enhances adipogenesis and signaling pathways involving PPARγ and functions as a positive regulator of adipocyte differentiation (Yu et al., 2016). Cheng et al. (2018) showed that the expression of the anti-ALDH2 antibody was enhanced in GO patients and decreased in normal controls. In addition, anti-ALDH2 antibody levels are strongly correlated with the disease activity of GO (Cheng et al., 2018; Lin, 2024). The potential involvement of Wnt signaling dysregulation has been implicated in GO pathogenesis through the regulation of adipogenesis (Tao et al., 2017). The downregulation of secreted frizzled-related protein 4 (SFRP4), a secreted antagonist of the Wnt signaling pathway, was found in orbital fat from GO patients compared to normal controls (Ezra et al., 2012).

The protein encoded by spermidine/spermine N1 (SAT1) belongs to the acetyltransferase family and is a rate-limiting enzyme in the catabolic pathway of polyamine metabolism. It catalyzes the acetylation of spermidine and spermine and is involved in the regulation of the intracellular concentration of polyamines and their transport out of cells. An increased concentration of spermine was found in the tears of patients with active GO compared to inactive GO (Billiet et al., 2022). The increased synthesis of spermine could be related to the overstimulation of Graves’ autoantibodies and could play a proliferative role at the origin of the increased myogenesis and adipogenesis observed in GO. The suppressor of cytokine signaling (SOCS) proteins are key regulators of immune responses, and SOCS3 functions predominantly as a negative regulator of cytokines that activate the JAK-STAT3 pathway, which participates in the regulation of key biological processes, including cell proliferation, differentiation, and apoptosis (Jiang et al., 2018). SOCS-3 also binds to the insulin growth receptor 1 (IGF1R) and may be a direct substrate for the receptor tyrosine kinase. IGF1R pathways play a critical role in the pathogenesis of GO, and teprotumumab, a human monoclonal anti-IGF1R blocking antibody, has been approved by the FDA for the treatment of patients with GO, specifically in reducing proptosis (Kahaly et al., 2021).

Oxysterols are metabolites derived from cholesterol oxidation and have been implicated in the pathogenesis of several diseases (Poli et al., 2013). These metabolites bind to liver X receptors (LXRs) and retinoic acid receptor-related orphan receptors (RORs), which are members of the nuclear receptor family of transcription factors and modulate gene expression involved in inflammatory and autoimmune processes (Duc et al., 2019). RORγT is a transcription factor involved in Th17 cell development (Ivanov et al., 2006). Several studies have suggested the involvement of pathogenic Th17 cells in GO (Jiang et al., 2022). It is proposed that the interplay between Th17 cells and OFs promotes orbital inflammation and fibrosis, and it strengthens Th17-OF communication via augmented costimulatory molecules (Fang et al., 2017). Hypercholesterolemia is a novel risk factor for GO, with both total high- and low-density lipoprotein cholesterol levels having recently been found to be associated with the presence of GO (Sabini et al., 2018; Lanzolla et al., 2018). This association is supported by a phase-2 randomized clinical trial involving 88 patients with active moderate-to-severe GO and increased low-density lipoprotein cholesterol levels, which demonstrated that adding atorvastatin to intravenous glucocorticoids led to a better treatment outcome than when using intravenous glucocorticoids alone (Lanzolla et al., 2021). The link between hypercholesterolemia and GO may reflect an altered inflammatory state in hypercholesterolemia (Ridker and JUPITER Study Group, 2003; Fonseca and Izar, 2009). Hence, the effect of statin on GO may go beyond lowering cholesterol levels with its pleiotropic effects on the adipogenesis of OFs and immunomodulatory actions (Lanzolla et al., 2019). Taken together, we postulate that hypercholesterolemia contributes to the inflammatory milieu of GO via oxysterols. Statins, by lowering cholesterol levels, ameliorate the oxidative stress exerted by this downstream metabolite.

We found S100A4 downregulation in both our transcriptome studies and tear protein analyses in patients with GO. In our earlier study investigating differences in tear protein profiles in different stages of TED, we observed a downward trend of S100A4 fold change with increasing severity of TED in both discovery and verification phase experiments (Chng et al., 2018). It is interesting that the same trend is observed in our RNA sequencing data from orbital fibroblasts derived from patients with GO when compared to normal controls, which suggests a common pathophysiology. The downregulation of this gene expression in GO was also confirmed via qPCR experiments in the current study. In addition, an interaction between S100A4 and several other genes implicated in early adipogenesis was evident based on our PPI network analysis. Although we postulate that the dysregulation trend of this protein in the tears may reflect limbal normality, S100A4, which belongs to the S100 superfamily of small Ca^2+^-binding proteins, also plays critical roles in the pathogenesis of autoimmune, fibrotic, and inflammatory disorders (Ambartsumian et al., 2019). Following the discovery of the correlation between the transcriptome and tear proteins, we explored protein–protein interaction networks between S100A4 and other candidate proteins based on our transcriptome analyses. Constructing protein interaction networks enables us to better understand disease mechanisms in GO through putative biological pathways. Such an approach has proven to be valuable in understanding the pathogenic mechanisms underlying other autoimmune diseases such as systemic lupus erythematosus (SLE), multiple sclerosis (MS), and type 1 diabetes (Safari-Alighiarloo et al., 2014). Notably, the candidate proteins from our PPI analysis revealed largely immune-mediated mechanisms with the involvement of the JAK-STAT3 pathway, interferon lambda receptor 1 and interferon alpha and beta receptor subunit 1 (IFNLR1 and IFNAR1), interleukin-10 receptor alpha and beta subunits (IL10RA and IL10RB, respectively), Annexin A2 (ANXA2), etc., some of which were found to be dysregulated in orbital tissues in patients with GO (Matheis et al., 2015; Falkowski et al., 2020; Gianoukakis et al., 2008; Wakelkamp et al., 2003). In addition, cross-interactions between S100A4 and proteins (based on our DEG results) previously noted to be dysregulated in GO, such as MAP2K6, SOCS3, and ALDH2, were also elucidated from this analysis.

There are limitations to our study. The small study sample size was due to strict inclusion and exclusion criteria for cases recruited for the study. Despite a small sample size, this study used next-generation sequencing technology and obtained robust results. In addition, the results of the discovery phase experiments were confirmed by the validation phase experiments, which increases the reliability of the results generated. Although the tear protein profile study was carried out at a different time point, the study was conducted in the same institution with similar patient profiles. Despite a small dataset, we observed a similar downward trend of S100A4 levels in both our transcriptome studies and tear protein analyses in patients with GO. This suggests that in the same disease milieu, whether at the orbital fibroblast or tear level, S100A4 potentially plays a role in the disease manifestations.

There were two patients with hyperlipidemia on statin treatment recruited for the study (one in the discovery phase and one in the validation phase), and recent studies suggest that statin may have beneficial effects on TED. Whether this has an implication on adipogenesis or gene expression is unknown. Although it may be difficult to control all lifestyle factors, we acknowledge that comorbidities, medication history, or lifestyle factors are potential confounders for our results and should be further explored in future studies.

Recent studies have demonstrated the utility of single-cell RNA sequencing (scRNA-seq) as a powerful tool to study cellular heterogeneity and complex cellular events. Notably, Li et al. (2022) utilized scRNA-seq to create comprehensive transcriptional atlases of the cellular components in the orbital connective tissue (OCT) from healthy controls (HCs) and individuals with GO although several potential limitations still exist. Epigenetic profiling using single-cell ATAC-seq could also provide further resolution of the transcriptomic patterns found in our study. Although these techniques were not explored in our study, they would have been helpful in validating our findings.

## Conclusion

The value of the transcriptome approach utilized in our study lies in its ability to generate candidate genes and pathways for further studies. Our study identified several DEGs and potential gene pathways in GO patients, which concurred with the results of other studies. This not only increased our understanding of the disease pathogenesis but also elucidated potential drug targets (e.g., teprotumumab and statins) and biomarkers (e.g., anti-ALDH2 antibody). The results of the PPI network analysis propose a biological explanation of this phenomenon, with potential crosstalk with other gene pathways discovered by our transcriptome analysis. Importantly, our study also identified S100A4 downregulation in both transcriptome studies and tear proteome analyses. Tear S100A4 may serve as a biomarker for the propensity to develop TED in patients with AITD before clinical manifestation and should be confirmed in future studies.

## Data Availability

The data presented in the study are deposited in the European Nucleotide Archive (ENA) repository, accession number PRJEB73574. The data can be found: https://www.ebi.ac.uk/ena/browser/view/PRJEB73574.
